# Improvement of Fatigue Life of GH3039 Superalloy by Laser Shock Peening

**DOI:** 10.3390/ma13173849

**Published:** 2020-08-31

**Authors:** Yang Tang, MaoZhong Ge, Yongkang Zhang, Taiming Wang, Wen Zhou

**Affiliations:** 1 School of Materials Engineering, Jiangsu University of Technology, Changzhou 213001, China; YangTang@jsut.edu.cn (Y.T.); WenZhou@jsut.edu.cn (W.Z.); 2 School of Electromechanical Engineering, Guangdong University of Technology, Guangzhou 510006, China; zykseu@163.com; 3 Development department, China Aero Engine Group Changzhou Lanxiang Machinery Co., Ltd., Changzhou 213021, China; suncanmachine@163.com

**Keywords:** laser shock peening, GH3039 superalloy, microstructure, fatigue life

## Abstract

In order to improve fatigue life of GH3039 superalloy, GH3039 superalloy sheets were treated by laser shock peening (LSP). The microstructure of GH3039 superalloy before and after LSP was characterized using an optical microscope, transmission electron microscope (TEM), and X-ray diffractometer. The fatigue life of the samples with and without LSP was investigated by fatigue experiments. Moreover, surface profile and residual stress were also examined. Experimental results indicated that the grains in the surface layer of the LSP sample were remarkably refined and reached the nanometer scale. The average surface roughness increased from 0.024 μm to 0.19 μm after LSP. The average fatigue life of the laser treated samples was 2.01 times larger than that of the untreated specimens. Additionally, mathematical statistical analysis confirms that LSP has a significant influence on the fatigue life of GH3039 superalloy. The improvement of fatigue life for the laser processed GH3039 superalloy was mainly attributed to compressive residual stress and grain refinement generated by LSP.

## 1. Introduction

As a solid solution strengthened austenitic alloy, the nickel-based superalloy GH3039 has been widely used for critical parts of aeroengines due to its excellent mechanical properties, good anti-oxidation, and high corrosion resistance [1,2,3]. With the increase of aero-engine thrust, GH3039 superalloy is required to withstand higher stresses and operating temperatures. In order to decrease damage of parts under extreme working conditions, surface treatment technologies, such as shot peening [4], cavitation peening [5], deep cold rolling [6], and laser shock peening (LSP) [7], are utilized to improve the service life of key parts.

Comparing with other surface treatment methods, LSP, as an innovative surface processing technology, can significantly improve fatigue resistance of metallic materials by introducing deeper residual compressive stress layer and ultrahigh strain rate plastic deformation [8]. Correa et al. [9] found that the fatigue life of the laser peened Al2024-T351 samples was about 3.5 times larger than the un-peened specimens. Zhou et al. [10] indicated that the fatigue life of the laser treated TC4 specimens increased by 107.86% relative to the original specimens. Moreover, LSP was employed to realize the microstructure change in the surface layer of the metallic materials. Ren et al. [11] confirmed that LSP could induce surface nanocrystallization of AZ91D magnesium alloy by severe plastic deformation. Lu et al. [12] found that the refined grains in the surface layer of pure titanium were created by LSP-induced ultrahigh plastic strain. Recently, some studies on surface morphologies, microstructural changes in the surface layer, and mechanical properties of superalloys treated by LSP have been performed. Ren et al. [13] revealed the remarkable influence of LSP on surface roughness, micro-hardness and microstructure of iron-based alloy GH2036 by experiment and simulation. Micheal Kattoura et al. [14] demonstrated the influence of LSP on microstructure, residual stress and the fatigue life of ATI Allvac Company(ATI) 718 Plus alloy. Saeid Amini et al. [15] verified the effects of laser beam diameter, number of laser impacts and laser intensity on fatigue life and hardness of Incoloy 800 superalloy. Zhou et al. [16] studied the influence of LSP on the high-temperature fatigue life of GH4133B superalloy turbine blades. Cao et al. [17] investigated the effects of LSP on microstructure and hot corrosion resistance of nickel-based superalloy GH202. However, research about GH3039 superalloy subjected to LSP is relatively few. As one of the heat-stable materials used in aeroengines, the turbine guider made of GH3039 superalloy is sometimes faced with premature fatigue fracture during its service, which not only seriously affects the safe flight of aircraft, but also greatly shortens the maintenance cycle and increases the cost of aeroengine manufacturing enterprises. In order to solve the problem of short fatigue life of GH3039 superalloy turbine guiders without changing the material and structure, it is urgent to improve the fatigue behavior of this superalloy by LSP.

To evaluate the influence of LSP on the fatigue life of GH3039 superalloy, a comparative analysis of the phase composition, residual stress state, microstructure, surface roughness, fatigue life and fatigue fracture morphology of GH3039 superalloy was conducted before and after LSP. Furthermore, mathematical statistics was utilized to analyze the fatigue life of GH3039 superalloy with and without LSP, and the strengthening mechanism of GH3039 superalloy after LSP was discussed. The objective of this paper is to provide a technological and theoretical basis for LSP technology application in aeroengine.

## 2. Materials and Methods

### 2.1. Materials and the Specimen Preparation

**Figure 1:**
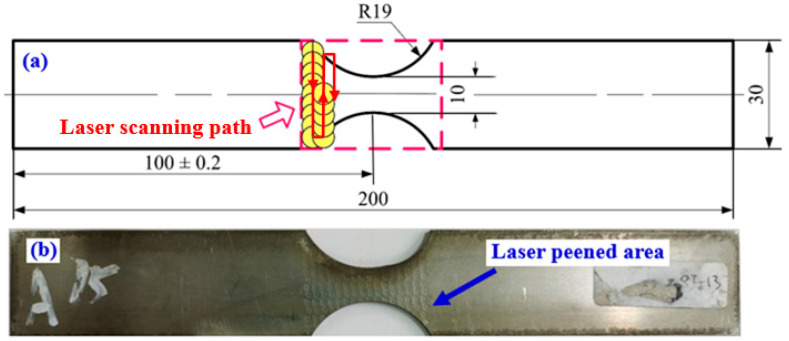
(**a**) Dimensions of fatigue sample and (**b**) LSP sample.

The experimental material is an aerial cold rolled 3039 superalloy sheet with 2 mm in thick. The chemical composition of GH3039 superalloy is shown in Table 1. Its ultimate stress and yield stress are 763 MPa and 520 MPa, respectively. The GH3039 superalloy fatigue specimens are cut by means of electric spark machining, which are presented in Figure 1. It should be noted that the length direction of all fatigue specimens is parallel to the rolling direction. Moreover, an arc of radius 19 mm of fatigue samples was machined after LSP. Fatigue specimens are divided into two groups: one is the as-received sample, and the other is laser treated sample. Each group contains six specimens. Before LSP, all samples were cleaned in acetone and then dried by hot-air.

### 2.2. LSP Treatment

LSP was conducted on a Procudo 200 (LSP Technologies, Dublin, OH, USA) laser peening system operating at 10 Hz repetition rate with a wavelength of 1053 nm and the pulse duration of about 20 ns. The laser pulse energy, the laser beam diameter and the overlapping rate were 8 J, 3 mm and 50%, respectively. According to the above laser processing parameters, the calculated laser intensity for each pulse was 5.66 GW/cm^2^. A flowing water curtain was selected as the transparent overlay with a thickness of about 2 mm, and a black tape was used as the opaque overlay with a thickness of 0.1 mm. Considering real geometric structure of aeroengine turbine guider, fatigue samples were only treated by one-sided laser shock peening and the sample treated area was 33 mm × 30 mm. The laser scanning path was perpendicular to the rolling plan directions (as shown in Figure 1a).

### 2.3. Measurement Instruments and Methods

Cross-sectional microstructure of GH3039 superalloy before and after LSP was observed using Axio Imager Mzm optical microscope (Carl Zeiss Microlmaging GmbH, Göttingen, German). The grain size of sample was examined using Image-Pro Plus 6.0 analysis software (Media Cybernetics, Rockville, MD, USA). The microstructures in near-surface layer of sample after LSP were detected using a JEM-2100 electron microscope (JEOL Ltd., Akishima, Japan). Cross-sectional TEM thin foils (10 μm × 2 μm × 0.1 μm) were cut directly from the laser peened sample by a focused ion beam (FIB). The XRD qualitative analysis was carried out by X’ Pert PRO X-ray diffractometer (Malvern Instruments, Malvern, UK) with operating voltage at 40 kV and current at 40 mA using Cu target radiation. The range of diffraction angle was selected from 20° to 80°, and the scanning speed was 2°/min. Surface roughness (Ra) of all samples before and after LSP was examined by NANOVEA PS50 (Nanovea, Salt Lake, UT, USA) three-dimensional non-contact profilometer. X-350A X-ray diffractometer was employed to detect the residual stress with the sin2 ψ method. Cr target radiation was employed and the (311) diffraction plane was selected. Stress constant K was –322 MPa/(°). The operating voltage and current of the X light tube were 18 kV and 4 mA, respectively. The scanning starting angle and ending angle were 148° and 158°, respectively. The scanning speed was 0.2°/min. The material in the surface layer of samples with and without LSP was removed layer by layer using an electropolishing method. The electrolyte consists of 5% nitric acid solution and 95% alcohol solution. The corrosion rate was about 0.3 μm/s each 1 cm^2^. In order to guarantee the reliability of the experimental results, test must be repeated at least three times for each condition. The results reported are the average of the repeated test.

### 2.4. Fatigue Experiment

**Figure 2:**
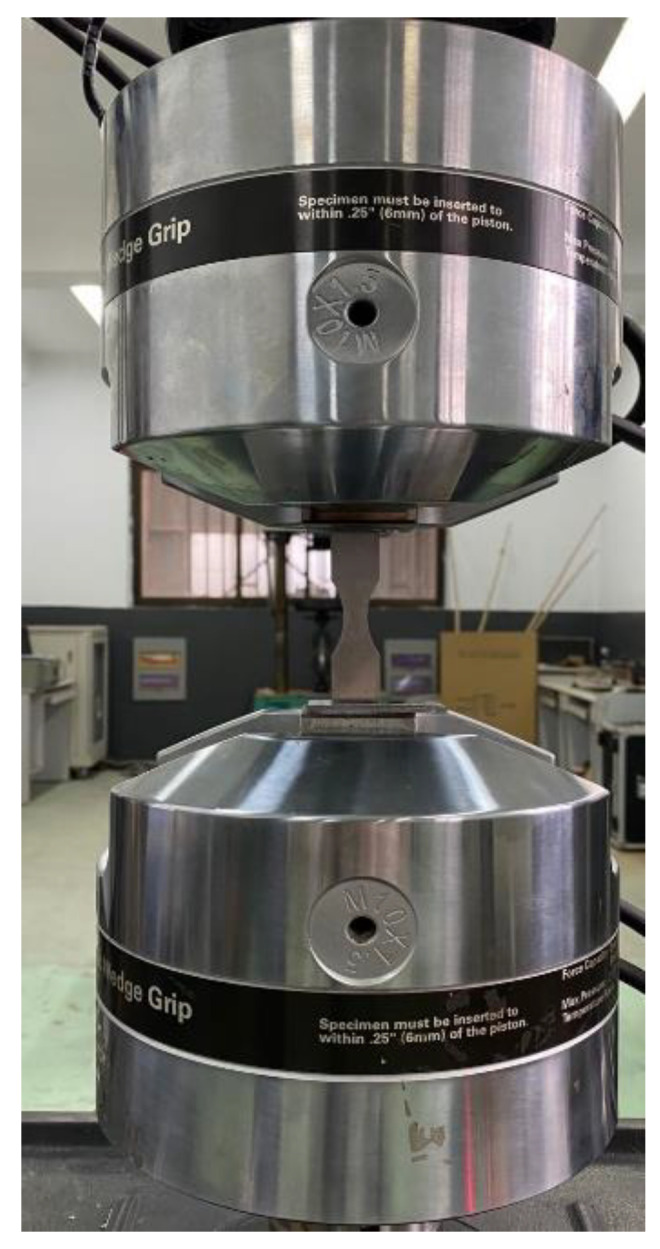
The photograph of the fatigue testing on GH3039 superalloy specimen.

Fatigue experiments were performed on MTS Landmark 370.10 servo-hydraulic test system (MTS Systems Corporation, Eden Prairie, MN, USA) at room temperature and relative humidity of 50–60% in the air. A 20 Hz sinusoidal loading scheme was utilized in the experiments and the stress amplitude remained constant. The stress ratio R was 0.1 and the maximum externally applied stress was 686.7 MPa. The microstructure of fatigue fracture was observed using a ∑IGMA500 scanning electron microscope. A photograph of the fatigue testing is shown in Figure 2.

## 3. Results and Discussion

### 3.1. Phase Analysis

**Figure 3:**
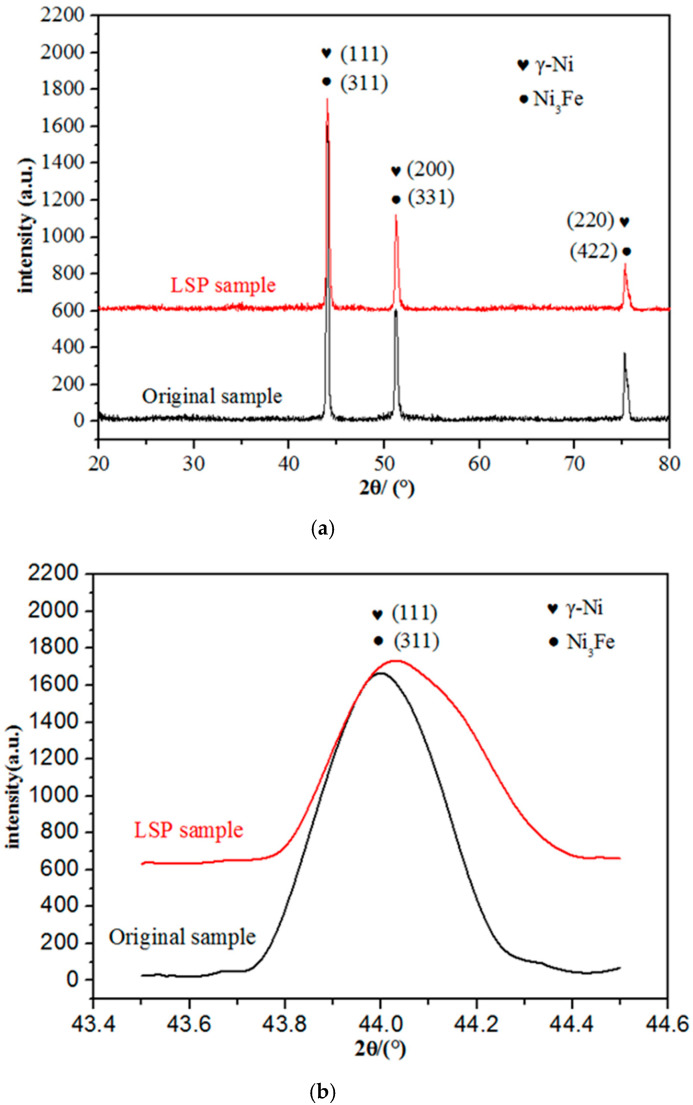
(**a**) XRD patterns of GH3039 with and without LSP treatment; and (**b**) Local magnified graph in the 43.5°–44.5° range of GH3039 XRD patterns with and without LSP treatment.

XRD patterns of the GH3039 superalloy with and without LSP treatment are displayed in Figure 3. From Figure 3a, we can clearly see that the surface of the GH3039 superalloy mainly consists of a γ phase (main phase) and a γ′ phase (strengthening phase). The number of diffraction peaks of the GH3039 superalloy does not change before and after LSP treatment, which indicates that neither new phase nor phase transition has occurred. In Figure 3b, it can be found that the diffraction peaks intensity of LSP sample is apparently lower than that of the as-received sample. In addition, all diffraction peaks are shifted to the right after LSP. This phenomenon may be attributed to compressive residual stress induced by LSP and it is in good agreement with the Bragg diffraction equation. When compressive residual stress exists in the sample, the interplanar spacing will reduce. The diffraction peak will move to the right when Bragg diffraction occurs, and the magnitude of the movement is related to the stress. Similar results were presented in different works using LSP [7,18]. According to Jade 6, the FWHM of diffraction peak (111) increased from 0.139 to 0.252 radian. The broadening of diffraction peak after LSP may be attribute to grain refinement, crystal lattice distortion and micro-strain development caused by laser-generated high-pressure shock wave [19,20]. This is well consistent with the phenomenon found in other severe plastic deformation (SPD) methods such as ultrasonic shot peening [21].

### 3.2. Microstructure

**Figure 4:**
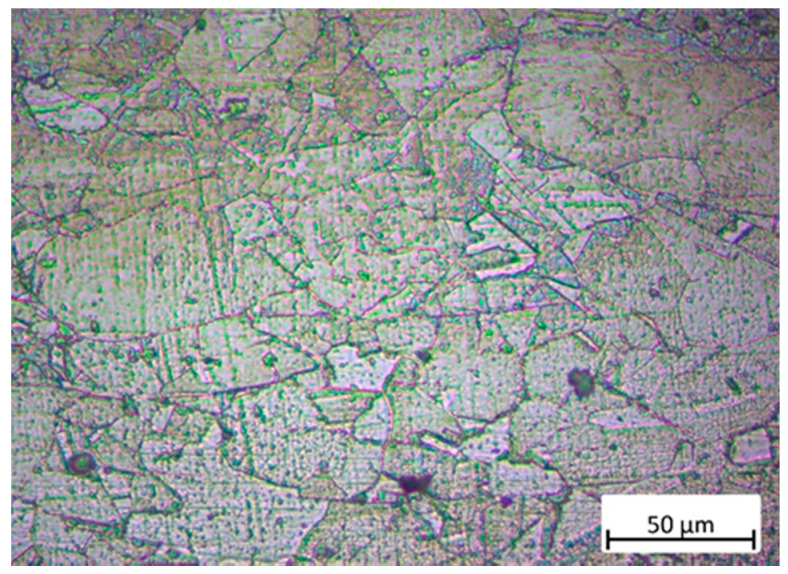
Cross-sectional optical micrograph of the original GH3039 superalloy sample.

Figure 4 shows cross-sectional optical micrograph of the original GH3039 superalloy sample. From Figure 4, we can find that the grains of the original sample are distributed very unevenly, and there are some strengthening phases dispersed in the γ-austenite substrate or at the grain boundaries. Moreover, the average grain size is around 35.3 μm according to Image-Pro Plus 6.0 analysis software (Media Cybernetics, Rockville, MD, USA).

**Figure 5:**
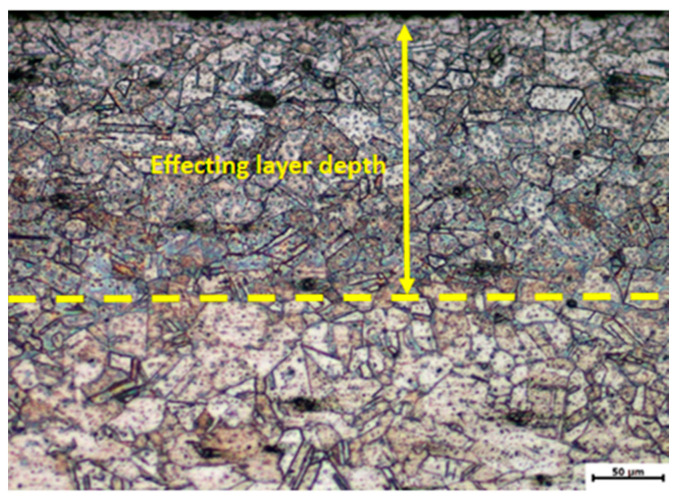
Cross-sectional optical micrograph of the laser treated GH3039 superalloy sample.

Figure 5 displays the cross-sectional microstructure of GH3039 superalloy after LSP. It should be noted that the upper surface is the laser peened surface. Obviously, there is a boundary (marked in yellow dash line) between severe plastic deformation zone and the substrate in the cross section, and the depth of severer plastic deformed layer can be identified to be about 195 μm from this figure. The grain size above the yellow line is smaller than that below the yellow line, and the average grain size in the severer plastic deformation zone is around 17.6 μm. Compared with the original sample (as shown in Figure 4), the grain size decreases significantly after LSP treatment. Additionally, the color above the yellow line is obviously darker than that below the yellow line. This is due to laser-induced severe plastic deformation leading to an evident increase in precipitates inside the grains compared to the untreated sample. From Figure 5, it can be found that there is a gradient structure generated in the deformed surface layer of GH3039 superalloy after LSP treatment, and grain boundaries could not be identified in the top surface layer and the grain size increases with the depth from the laser processed surface. This is due to the fact that, when the high-pressure shock wave generated by LSP penetrates into the sample, the decay of shock wave pressure increases gradually with the propagation distance increasing, and consequently, the plastic deformed level also decreases with the increment of the depth, which means the corresponding deformation strain and strain rate levels reduce gradually.

**Figure 6:**
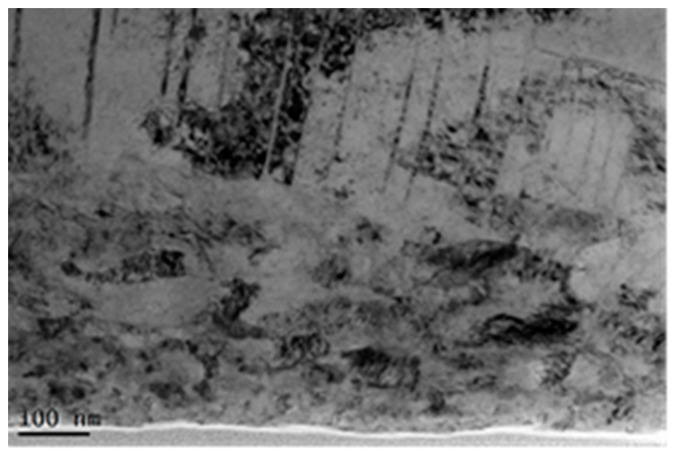
The cross-sectional TEM image of the laser treated GH3039 superalloy.

The cross-sectional TEM micrograph of the laser treated GH3039 superalloy is shown in Figure 6, where the lower surface is the laser processed surface. The evidences of severe plastic deformation can be observed in the very top surface layer. Close to the top surface, some grains are refined to nanometer level and some nano-grain boundaries are clearly observed. A large number of dislocation tangles, dislocation cells, and dislocation walls can be seen away from the laser treated surface. Dislocation distribution is very uneven, and the dislocation density is very high in some local area. With the further increase in the distance away from the laser processed surface, most original grain has been subdivided into many band-shaped substructures with a width of about 25–110 nm along the direction of short axis, which are subdivided by dislocation pile-ups.

**Figure 7:**
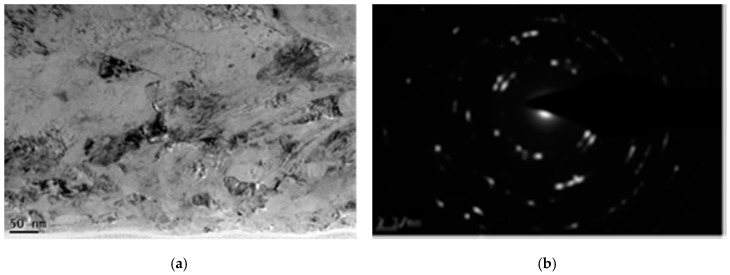
The locally enlarged cross-sectional TEM image of the laser treated GH3039 superalloy (**a**) and the corresponding selected area electron diffraction pattern (**b**).

Figure 7 shows the locally enlarged cross-sectional TEM image of the LSP sample. In Figure 7a, the size of nanocrystals in the very top surface layer is around 12–75 nm. Some nanocrystalline boundaries are clear and complete. Furthermore, there are dislocation tangles in the nano-grains. The corresponding selected area electron diffraction pattern, as shown in Figure 7b, exhibits discontinuous diffraction spots elongated into a circular shape, demonstrating that the grains of the laser processed sample have been refined obviously and subdivided into nanocrystals with random orientation.

**Figure 8:**
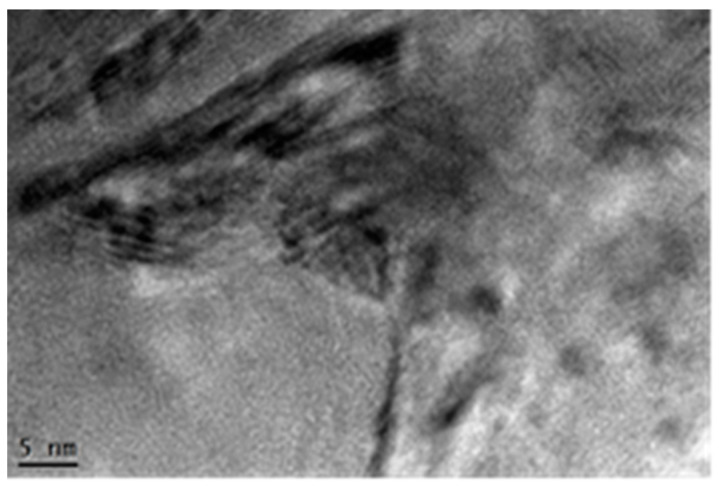
HRTEM image in the top surface layer of GH3039 superalloy after LSP.

Figure 8 gives the corresponding high-resolution transmission electron microscopy (HRTEM) image in the top surface layer of the laser treated GH3039 superalloy. From Figure 8, the nanocrystalline with clear boundary was observed. In the interior of the same grain, there is the obvious contrast variation, which could be caused by the residual stress, dislocation pile-ups, and the crystalline lattice distortion. Moreover, there are high density dislocations and dislocation pile-ups in the nano-grains.

According to Figure 4, Figure 5, Figure 6, Figure 7 and Figure 8, the grain refinement mechanism of GH3039 superalloy after LSP may be described as following: At the beginning of deformation, i.e., close to the substrate, the high-density dislocations and the band-shaped substructures are produced due to the small plastic deformation created by LSP. With the increase of plastic deformation, dislocation tangle, dislocation wall and dislocation cell would be formed. When the increase of plastic deformation to a certain extent, the dislocation tangles would transform into sub-grain boundaries with low angle through continuously absorbing new dislocations for minimizing the total energy of system. With further increase of plastic deformation, the sub-grain boundaries would gradually transform into the grain boundaries high angle. Finally, the nano-grains with random orientation are generated in the top surface layer. Similar results were presented in different works using LSP [14,22].

### 3.3. Surface Roughness Analysis

**Figure 9:**
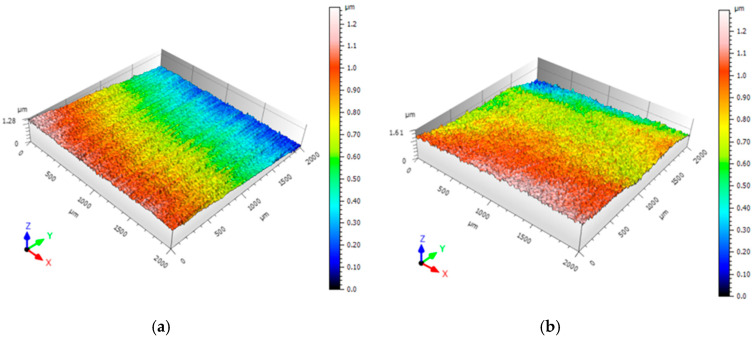
Three-dimensional surface topographies of the GH3039 superalloy before and after LSP: original sample (**a**) and LSPed sample (**b**).

Surface roughness has a significant influence on metallic material fatigue property. Usually, the increment of surface roughness can lead to the decrement of fatigue resistance. Figure 9 shows the three-dimensional surface topographies of GH3039 superalloy samples before and after LSP. It should be noted that the surface roughness Ra is an average value of all points selected by the analysis software of three-dimensional non-contact profilometer. The average surface roughness Ra for the original and the laser treated sample are respectively 0.024 μm and 0.19 μm, which indicates that LSP could lead to a slight increase of the surface roughness for GH3039 superalloy samples. Similar results were presented in different works using LSP [23,24,25].

### 3.4. Residual Stress Analysis

**Figure 10:**
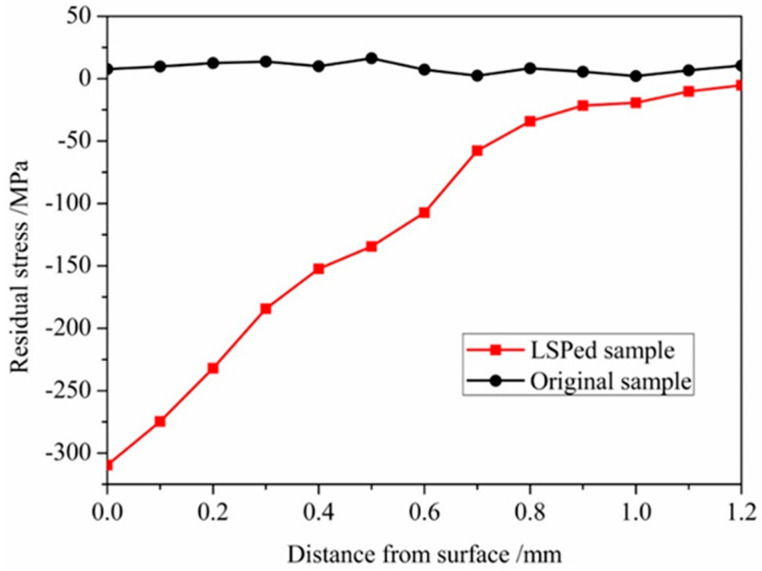
Residual stress distribution in depth of GH3039 sample before and after LSP treatment.

When high-pressure shock wave induced by LSP acts on the target surface, severe plastic deformation will occur in the target surface layer. The plastic deformation region is confined by the surrounding elastically deformed material, and results in high-amplitude compressive residual stress that restrains the crack initiation and reduces crack growth rate [8]. The residual stress variation curves along the depth direction of GH3039 superalloy sample with and without LSP treatment are depicted in Figure 10. One measurement was conducted for each 100 μm along the depth of the sample. Before LSP, there are residual tensile stresses in the range of from 5 to 28 MPa generated by cold rolling in the surface layer of GH3039 superalloy. After LSP, the residual stress state of specimens was changed from tensile to compressive stress. For laser processed sample, the maximal compressive residual stress exits in the sample surface, and it reaches to –316.5 ± 16 MPa. From Figure 10, we can find that the compressive residual stress reduces gradually with the increment of the depth, and the plastic deformation layer tends to about 1.2 mm. This is attribute to the fact that along the laser-induced shock wave propagation direction, most of the shock wave energy is gradually converted into plastic deformation energy, which leads to the decay of shock wave pressure with the increase of the propagation distance. The lower the shock wave pressure is, the lower the plastic deformation degree is, and the smaller the compressive residual stress is. Similar results were presented in different works using LSP [7,16,18].

### 3.5. Fatigue Life Analysis

The fatigue test results of the GH3039 superalloy samples before and after LSP are presented in Table 2. Under the same applied load, the average fatigue life of the original samples and the laser treated samples is 27,633 and 83,266, respectively. It indicates that the average fatigue life of the LSP samples is 2.01 times larger than that of the untreated specimens. The fatigue life mainly depends on the crack initiation and crack growth rate. The above test results indicate that LSP not only can achieve grain refinement and generate compressive residual stress in the surface layer of specimen, but also increase surface roughness of specimen. Laser-induced grain refinement is very beneficial to increase the fatigue life of samples. On the one hand, refined grains can restrict the initiation of cracks by improving the strength of sample. On the other hand, refined grains can inhibit crack growth by increasing grain boundaries. Meanwhile, laser-induced compressive residual stress cannot only prevent the initiation of cracks by balancing a part of working stress, but also block the growth of cracks by enhancing the threshold of crack propagation. In addition, fatigue samples are very sensitive to stress concentration, so the increase of surface roughness caused by LSP will reduce the fatigue life of GH3039 superalloy samples. However, laser-induced microstructure changes and compressive residual stresses are the main factors affecting the fatigue life of the GH3039 superalloy.

### 3.6. Mathematical Statistical Analysis

The fatigue life of GH3039 superalloy specimen before and after LSP treatment, denoted by ξ and η, respectively, are defined as two random variables during hypothesis testing. It is assumed that the random variable ξ and η have a normal distribution, i.e., $\xi \sim N\left(\mu_{1},\sigma_{1}^{2}\right)$, $\eta \sim N\left(\mu_{2},\sigma_{2}^{2}\right)$. However, mean $\mu_{1}$ and $\mu_{2}$ and standard deviation $\sigma_{1}$ and $\sigma_{2}$ are unknown. When the significance level α is 0.05, the following hypothesis test can be conducted.

(1) $$H_{0}:\sigma_{1}^{2}=\sigma_{2}^{2}$$

(2) $$H_{1}:\sigma_{1}^{2}\neq \sigma_{2}^{2}$$

The sample mean and the corrected sample variance of the GH3039 superalloy sample with and without LSP treatment are calculated as follows:

(3) $$n_{1}=6,\;\bar{x}=84432,\;{s_{1}^{*}}^{2}=2316431$$

(4) $$n_{2}=6,\;\bar{y}=27633,\;{s_{2}^{*}}^{2}=481526.8$$

where $n_{1}$ and $n_{2}$ represent the number of the laser peened sample and the original sample respectively. $\bar{x}$ and $\bar{y}$ represent the average value of fatigue lives for the laser peened sample and the original sample respectively. ${s_{1}^{*}}^{2}$ and ${s_{2}^{*}}^{2}$ represent the corrected sample variance of the laser peened sample and the original sample respectively.

The value of the test statistic F can be defined as the formula:

(5) $$F={s_{1}^{*}}^{2}/{s_{2}^{*}}^{2}$$

Therefore: $F={s_{1}^{*}}^{2}/{s_{2}^{*}}^{2}=4.81$.

According to the quantile table of F distribution, it can be known that:

(6) $$F_{1-\frac{\alpha }{2}}\left(n_{1}-1,n_{2}-1\right)=F_{1-0.025}\left(5,5\right)=7.15$$

Because $F={s_{1}^{*}}^{2}/{s_{2}^{*}}^{2}=4.81<7.15=F_{1-\frac{\alpha }{2}}\left(n_{1}-1,n_{2}-1\right)$, the hypothesis $H_{0}$ is accepted, this would indicate the variance of the two groups of samples is equal, i.e., $\sigma_{1}^{2}=\sigma_{2}^{2}$.

When the values of $\sigma_{1}^{2}$ and $\sigma_{2}^{2}$ are unknown, but $\sigma_{1}^{2}$ is known to be equal to $\sigma_{2}^{2}$, the following hypothesis test can be carried out at the significance level α of 0.05.

(7) $$H_{0}:\mu_{1}-\mu_{2}=0$$

(8) $$H_{1}:\mu_{1}-\mu_{2}\neq 0$$

According to the sample mean and the corrected sample variance, the value of the test statistic t can be obtained by the following equation:

(9) $$t=\frac{\left|\bar{x}-\bar{y}\right|}{\sqrt{\frac{\left(n_{1}-1\right){s_{1}^{*}}^{2}+\left(n_{2}-1\right){s_{2}^{*}}^{2}}{n_{1}+n_{2}-2}\times }\sqrt{\frac{1}{n_{1}}+\frac{1}{n_{2}}}}$$

Therefore, *t* = 81.4.

According to the quantile table of T distribution, it can be known that:

(10) $$t_{1-\frac{\alpha }{2}}\left(n_{1}+n_{2}-2\right)=t_{1-0.025}\left(10\right)=2.2281$$

Because $t=81.4>2.2281=t_{1-\frac{\alpha }{2}}\left(n_{1}+n_{2}-2\right)$, the hypothesis $H_{0}$ is rejected, this would indicate that there is a significant difference in fatigue life of GH3039 superalloy between laser treated samples and base material samples, and further confirm that LSP has a significant influence on the fatigue life of GH3039 superalloy.

### 3.7. Fracture Morphologies Analysis

**Figure 11:**
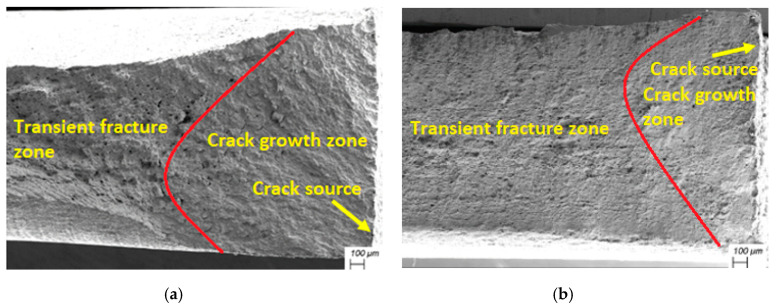

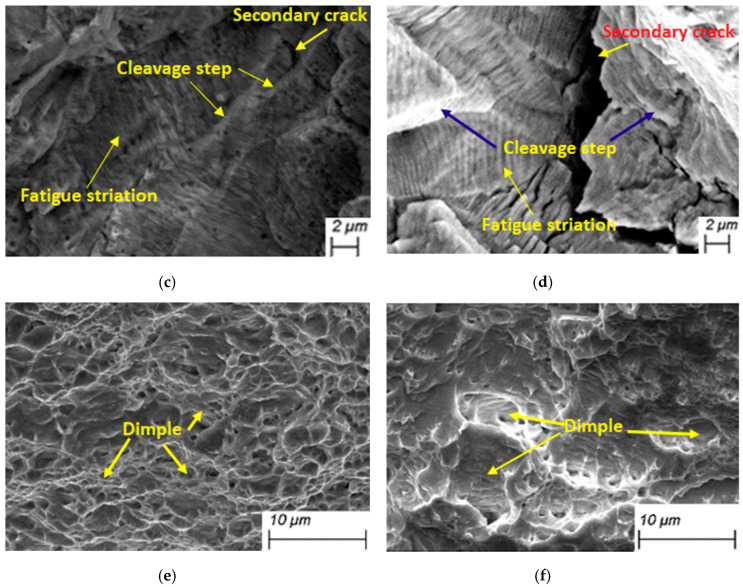
Fracture morphologies of GH3039 superalloy before and after LSP: Macroscopic fracture morphologies before (**a**) and after LSP (**b**); Fatigue crack growth area morphologies before (**c**) and after LSP (**d**); Transient fracture area morphologies before (**e**) and after LSP (**f**).

Fatigue fracture morphologies of GH3039 superalloy before and after LSP are shown in Figure 11. Macroscopic fatigue fracture morphologies without and with LSP treatment are presented in Figure 11a,b, respectively. Figure 11a,b indicates that the degree of section shrinkage of laser treated sample is significantly greater than that of the original GH3039 superalloy specimen, which is attribute to grain refinement caused by LSP. Compared with coarse grains, LSP-induced grain refinement can increase the number of grains per unit volume. When subjected to the same applied load, the deformation can be shared by more grains to make the deformation more uniform, then improving the plasticity of the material. In addition, the crack source is generated on the side of the arc region for the specimen with and without LSP. Fatigue fracture morphologies in the crack growth area without and with LSP treatment are given in Figure 11c,d, respectively. It can be seen from Figure 11c,d that there are obvious cleavage steps and fatigue strips, which means that the fracture modes of both the original sample and the laser peened sample belong to cleavage fracture in the crack growth zone. Apparently, the distance between adjacent fatigue strips of the original GH3039 superalloy sample is much larger than that of LSP specimen. This indicates the fatigue propagation rate of the laser peened specimen is lower than that of the original specimen, which may be due to the inhibiting effect of high-density dislocations, new grain boundaries and compressive residual stress. In addition, there are secondary cracks in both the base material and the laser impact sample. Fatigue fracture morphologies in the transient fracture area without and with LSP are presented in Figure 11e,f, respectively. A large number of dimples can be found in the transient fracture area of both different samples, which indicates the ruptured mode in this zone is all ductile fracture. Furthermore, the size of dimples for the LSP sample is smaller than that of the original GH3039 superalloy specimen, which is attributed to the grain refinement caused by LSP.

### 3.8. Fatigue Resistance Mechanism of GH3039 Superalloy Subjected to LSP

According to the above experimental results, the strengthening mechanism of GH3039 superalloy after LSP can be concluded as following:

Firstly, when the shock wave with ultrahigh pressure over several GPa created through LSP acts on the sample surface, severe plastic deformation will occur in the sample surface layer, which can effectively activate dislocation slip, and then resulting in the evident increment of dislocation density and obvious grain refinement. Refined grains are helpful to increase the grain boundary area per unit volume and decrease the concentration of impurity elements at grain boundaries, which can effectively improve the slip deformation resistance and restrict or impede the generation of slip bands. In the process of loading, grain refinement can provide more grains to bear a certain amount of deformation, which can reduce the stress level leading to local cracking and make plastic deformation more uniform, thus effectively inhibiting crack initiation. In the phase of crack growth, when fatigue crack passes through grain boundary from one grain to another, the fatigue crack growth rate will be reduced due to the impeding of the grain boundaries. In the subsurface, high-density dislocations in grains can effectively restrict the plastic flow and prevent the fatigue crack propagation.

Secondly, LSP-induced compressive residual stress in the surface layer of GH3039 superalloy sample can obviously decrease the effective crack driving force by balancing a portion of working tensile stress acted on specimens during fatigue tests, which can remarkably improve fatigue strength and prevent fatigue crack initiation of GH3039 superalloy sample. Additionally, compressive residual stress can reduce the stress intensity factor range [25], and then inhibit fatigue crack growth of the laser treated GH3039 superalloy during fatigue tests.

## 4. Conclusions

In this study, LSP was employed to induce severe plastic deformation, generate surface nanostructure, refine grain, impart compressive residual stress, and improve the fatigue life of GH3039 superalloy. The main conclusions are as follows:

(1) The laser-induced severe plastic deformation varied the microstructure in the GH3039 superalloy surface layer. Nanometer grains with the size in the range of 12–75 nm could be generated in the surface layer of GH3039 superalloy. Dislocation glide is the major cause of grain refinement of GH3039 superalloy induced by LSP.
(2) Compressive residual stress to a depth of about 1.2 mm was introduced into the surface layer of GH3039 superalloy after LSP, and the maximum compressive residual stress –316.5 ± 16 MPa.
(3) The average fatigue life of GH3039 superalloy increased from 27,633 to 83,266 after LSP. The main reason for the improvement in fatigue life of GH3039 superalloy subjected to LSP is due to the combined effects of grain refinement and compressive residual stress that can delay crack initiation and propagation.

## Figures and Tables

**Table 1:** The chemical composition of GH3039 superalloy (wt %).

**C**	**Cr**	**Al**	**Ti**	**Mo**	**Nb**	**Fe**	**Si**	**S**	**Mn**	**P**	**Ni**
≤0.08	19–22	0.35–0.75	0.35–0.75	1.8–2.3	0.9–1.3	3	≤0.8	≤0.012	0.4	0.02	Bal.

**Table 2:** Fatigue lives of GH3039 superalloy before and after LSP.

No.	1	2	3	4	5	6
Original sample	27,599	28,049	26,817	27,623	28,712	26,998
LSP sample	85,493	84,329	82,198	83,547	82,771	81,259
